# PRMT4 promotes hepatocellular carcinoma progression by activating AKT/mTOR signaling and indicates poor prognosis

**DOI:** 10.7150/ijms.62467

**Published:** 2021-08-27

**Authors:** Peng Du, Kaifeng Luo, Guoyong Li, Jisheng Zhu, Qi Xiao, Yong Li, Xingjian Zhang

**Affiliations:** Department of General Surgery, The First Affiliated Hospital of Nanchang University, Nanchang, Jiangxi 330006, China.

**Keywords:** PRMT4, hepatocellular carcinoma, progression, AKT/mTOR

## Abstract

**Background**: Protein arginine methyltransferase 4 (PRMT4) has been reported to play a role in several common cancers; however, the function and mechanism of PRMT4 in hepatocellular carcinoma (HCC) are not fully understood. This study aimed to investigate the role and mechanism of PRMT4 in the progression of HCC.

**Methods:** PRMT4 expression and clinicopathological characteristics were investigated using an HCC tissue microarray (TMA) consisting of 140 patient samples analyzed by immunohistochemistry. CCK-8, crystal violet and Transwell assays were used to determine cell proliferation, colony formation, migration, and invasion of HCC cell lines in which PRMT4 was overexpressed or downregulated. The underlying mechanism of PRMT4 function was explored by Western blot assays.

**Results:** PRMT4 was highly expressed in HCC tumor tissues compared to adjacent nontumor tissues. PRMT4 expression was significantly associated with alpha-fetoprotein levels, tumor size, satellite nodules, and microvascular invasion. Patients with higher PRMT4 expression had a shorter survival time and higher recurrence rate. Functional studies demonstrated that PRMT4 overexpression promoted HCC cell proliferation, migration, and invasion *in vitro*, while knocking down PRMT4 inhibited these malignant behaviors. Additional results revealed that PRMT4 promoted the progression of HCC cells via activation of the AKT/mTOR signaling pathway. Furthermore, inhibition of the AKT/mTOR signaling by MK2206 or rapamycin significantly attenuated PRMT4-mediated malignant phenotypes.

**Conclusions:** This study suggests that PRMT4 may promote the progression of HCC cells by activating the AKT/mTOR signaling pathway, which may be a valuable biomarker and potential target for HCC.

## Introduction

Hepatocellular carcinoma (HCC) is one of the most common malignant tumors with high morbidity and mortality. It is the sixth most common cancer and the third leading cause of cancer-related death worldwide 1. Multiple treatments, such as curative surgical resection, transarterial chemoembolization (TACE), radiotherapy, and molecular targeted therapy, are available for HCC patients; however, the prognosis for HCC patients remains unsatisfactory, mostly due to the high tendency of tumor recurrence and early vascular invasion 2, 3. Increasing evidence has revealed that HCC incidence is associated with multigene mutations 4, and molecular targeted therapy used to target these genes in advanced HCC patients is a novel treatment method that has exhibited favorable curative effects and significantly prolonged the patient's survival time 5. Therefore, understanding the molecular mechanisms underlying HCC development and finding new molecular targets for HCC treatment will greatly benefits these patients.

Protein arginine methylation is a widespread posttranslational modification that affects many cellular processes, including transcription, RNA splicing, DNA repair, cell signaling and cell fate determination 6. This posttranslational modification is mainly catalyzed by the protein arginine methyltransferase (PRMT) family. PRMTs, composed of nine members, can be divided into three types (I, II and III) based on their catalytic activity: type I PRMTs (PRMT1, 2, 3, 4, 6, and 8) are responsible for asymmetric dimethylarginine (ADMA) and monomethylarginine (MMA), type II PRMTs (PRMT5 and 9) generate symmetric dimethylarginine (SDMA) and MMA, and type III PRMT (PRMT 7) catalyze the formation of MMA 6, 7. Since the dysregulation of protein arginine methylation is closely related to the development of cancer, PRMTs, as novel anticancer drug development targets, have attracted increasing attention 6, 8, 9. To date, the oncogenic role of some PRMT isoforms, e.g., PRMT1, PRMT5, PRMT6, and PRMT9, has been reported in several tumor types 10-13. Specifically, PRMT4, which is also named coactivator-associated arginine methyltransferase 1 (CARM1), has been identified as an SRC-2/TIF2/GRIP1-binding protein 14. PRMT4 initially functions as a coactivator for many nuclear receptors and transcription factors and also acts as a coactivator of transcription factors in addition to nuclear receptors, such as p53 and NF-κB 15, 16. Multiple studies have indicated that PRMT4 plays critical roles in regulating a variety of cellular processes, such as transcription activation, RNA processing, tumorigenesis and cancer progression, cell growth/differentiation and apoptosis 17. Changes in PRMT4, mainly upregulation, are frequently reported in various types of human cancers, including breast cancer 18, lung cancer 17, colorectal cancer 19, and prostate cancer 20, which appears to promote cancer initiation, progression and metastasis. Moreover, PRMT4 elevation not only modulates the activity of cancer-related signaling pathways but also creates a favorable microenvironment for tumorigenesis and cancer progression 17. However, studies investigating PRMT4 function in the progression of HCC have been lagging, which leaves several questions about the role of PRMT4 unanswered.

This study focused on the function of PRMT4 in the progression of HCC. We demonstrated that PRMT4 was upregulated in human HCC tissues and promoted the proliferation, migration, and invasion of HCC cell lines *in vitro*. Additionally, we determined the underlying molecular mechanism by which PRMT4 promoted the progression of HCC cells by activating the AKT/mTOR pathway.

## Materials and Methods

### HCC tissue samples and tissue microarray analysis

Thirty pairs of HCC tissues and the corresponding nontumor liver tissue were obtained from patients who underwent liver resection at the First Affiliated Hospital of Nanchang University and the samples were subjected to quantitative real-time PCR analysis. To evaluate the expression pattern of PRMT4, we used a tissue microarray (TMA) containing 140 pairs of paraffin-embedded primary HCC tissues and adjacent nontumor liver tissues collected from the First Affiliated Hospital of Nanchang University between January 2012 and December 2016 and analyzed the relationship between the PRMT4 expression pattern and clinical features. Patients in the TMA cohort were followed up regularly, and the clinicopathological characteristics and survival information were recorded. This research was approved by the Ethics Review Committee of The First Affiliated Hospital of Nanchang University and was carried out in compliance with the Declaration of Helsinki. All participants signed an informed consent document before enrollment.

### Immunohistochemistry (IHC)

Paraffin-embedded tissue sections were deparaffinized and rehydrated before being subjected to endogenous peroxidase inhibition. The sections were rinsed three times in PBS (0.01 mol/l) and blocked with PBS (0.01 mol/l) containing 5% BSA and 0.3% Triton X-100 for 1 h. After incubation with anti-PRMT4 antibody (Proteintech, #55246-1-AP, 1:200) at 4°C overnight, the samples were incubated again with the secondary antibody at room temperature for 2 h. After being developed with 3,3'-diaminobenzidine (0.03%) and H_2_O_2_ (0.003%) in Tris-HCl (0.05 mol/l, pH 7.6), the extent of staining and the staining intensity were examined automatically by the Vectra 2 system (PerkinElmer, USA) and they were calculated using the H-score as previously described 18.

### Quantitative real-time polymerase chain reaction (qRT-PCR)

Total RNA was isolated from the clinical samples using TRIzol reagent (Invitrogen, Carlsbad, CA). Then, cDNA was synthesized from 1 μg of total RNA using the PrimeScript RT reagent Kit (DRR037A, TaKaRa, Japan). Real-time PCR was conducted using SYBR Premix Ex Taq (DRR081, Takara) on a LightCycler (Roche Diagnostics GmbH, Manheim, Germany). The PRMT4-specific primers were 5'-TCGCCACACCCAACGATTT-3′ (forward) and 5'-GTACTGCACGGCAGAAGACT-3′ (reverse), and the GAPDH primers were 5'-ATGACCCCTTCATTGACCTCA-3' (forward) and 5'-GAGATGATCACCCTTTTGGCT-3' (reverse). The relative expression level of PRMT4 was normalized to GAPDH using the 2^-ΔΔCt^ method 17.

### Cell culture

Human HCC cell lines (YY-8103, Hep3B, SNU-398, and Huh7) and the liver cell line THLE2 were purchased from the cell bank of the Shanghai Biology Institute, Chinese Academy of Science (Shanghai, China). All cells were grown in DMEM (Gibco) containing 1% penicillin/streptomycin (Sangong Biotech) and 10% FBS (Anlite) and maintained at 37 ℃ in a humidified incubator with 5% CO_2_.

### Cell transfection

The coding sequence for the full-length PRMT4 was cloned into the p23-ZsGreen plasmid (GeneChem, Inc., Shanghai) to generate the PRMT4 expression vector. Lentiviral short hairpin RNA (shRNA) targeting PRMT4 (GeneChem, Inc., Shanghai) was inserted into the pLKO.1-TRC vector. Cell transfection were performed with Lipofectamine 2000 Reagent (Invitrogen, USA) according to the manufacturer's instruction. YY-8103 cells were infected with p23-ZsGreen-PRMT4 lentivirus or negative control vector, and Huh7 cells were infected with pLKO.1-shPRMT4 or negative control duplex with a scramble sequence. Overexpressed and silenced cells were sorted using flow cytometry or selected by puromycin (4 µg/ml) for 4 days. Sequences of the interference targets are the followings: shPRMT4-1: 5'-GATCCCCCATGATGCAGGACTACGTGTTCAAGAGACACGTAGTCCTGCATCATGTTTTTC-3′ (forward) and 5'-TCGAGAAAAACATGATGCAGGACTACGTGTCTCTTGAACACGTAGTCCTGCATCATGGGG-3′ (reverse); shPRMT4-2: 5'-GATCCCCGGACATGTCTGCTTATTGCTTCAAGAGAGCAATAAGCAGACATGTCCTTTTTC-3′ (forward) and 5'-TCGAGAAAAAGGACATGTCTGCTTATTGCTCTCTTGAAGCAATAAGCAGACATGTCCGGG-3′ (reverse); Scramble shRNA: 5′-GATCCCCAATTGCCACAACAGGGTCGTGTTCAAGAGACACGACCCTGCCGTGGCAATTTTTTTC-3′ (forward) and 5′-TCGAGAAAAAAATTGCCACAACAGGGTCGTGTCTCTTGAACACGACCCTGCCGTGGCAATTGGG-3′(reverse).

### Western blotting

Cells were lysed in RIPA buffer (Thermo Scientific, Rockford, IL, USA) containing protease inhibitors on ice for 30 min. After centrifugation (10,000 g, 15 min, 4 ℃), the concentrations of protein samples were assessed using Bradford reagent (Sigma). Then, equal amounts of protein were separated by 10% SDS-PAGE and transferred onto PVDF membranes. After blocking with 5% skim milk for 2 h at room temperature, the membranes were incubated with the indicated primary antibodies overnight at 4°C. The primary antibodies used were as follows: PRMT4 (Proteintech, #55246-1-AP, 1:1000), AKT(Proteintech, #10176-2-AP, 1:1000), p-AKT (Proteintech, #66444-1-lg, 1:2000), mTOR(Proteintech, #66888-1-lg, 1:2000), p-mTOR (Proteintech, #67778-1-lg, 1:2000), 4E-BP1(Cell Signaling, #9644, 1:1000), p-4E-BP1(Cell Signaling, #2855, 1:1000), RPS6 (Proteintech, #14823-1-AP, 1:500), p-RPS6 (Proteintech, #29223-1-AP, 1:1000) and GAPDH (Proteintech, #10494-1-AP, 1:5000). Then, the membranes were incubated with corresponding horseradish peroxidase-conjugated secondary antibody (Proteintech, #SA00001-1/SA00001-2, 1:5000) for 2 h at room temperature. The immunoreactive protein bands were visualized with an ECL chemiluminescence system (GE Healthcare, Buckinghamshire, UK).

### CCK-8 assay

Transfected cells were seeded into a 96-well plate at a density of 1x10^4^ cells per well. After that, 10 μl of CCK-8 solution (Dojindo, Kumamoto, Japan) was added to the medium of each well at 0, 24, 48, and 72 h before the optical density (OD) was measured at 450 nm using a microplate reader (Molecular Devices, CA, USA).

### Crystal violet assay

The cells (1,000 cells/well) were grown in 6-well dishes containing culture medium and 10% FBS. The culture medium was changed every 3 days. Following 14 days of incubation, the cells were subjected to crystal violet staining and then photographed. Finally, OD values were measured at 600 nm using a microplate reader.

### Transwell assay

HCC cell invasion and migration analyses were carried out using a 24-well Transwell chamber (8-µm pore size; Corning, NY, USA). Briefly, a cell suspension (150 μL, 1 × 10^5^ cells) was positioned into the top chamber with FBS-free medium, and 500 μL medium with 10% FBS was positioned into the bottom chamber. For the invasion analysis, Matrigel (50 µl; BD Biosciences) was used to precoat the membrane surface. After incubation at 37°C for 24 h, the invading cells were fixed with methanol and then stained with crystal violet (0.1%) for 10 min at room temperature. The cells in 5 randomly chosen fields were counted under an optical microscope (Olympus Corp.) at ×200 magnification.

### Statistical analysis

All statistical analyses were processed using the SPSS 22.0 software package (SPSS Inc.) and GraphPad Prism 8.0 (GraphPad Software Inc.). All experimental results were independently repeated at least three times. Data are expressed as the mean ± standard deviation (SD). Categorical data were compared using Chi-square test. The OS and DFS rates of patients with HCC were estimated using Kaplan-Meier analysis. Independent predictors of OS and DFS were determined using univariate analysis and multivariate Cox regression analysis with stepwise selection. The significant prognostic factors upon univariate analysis (*P*<0.05) were subjected to multivariate analysis using the Cox proportional hazards regression model. Differences between groups were compared using Student's t-test or ANOVA followed by Tukey's post hoc test. Differences were considered to be significant at *P*<0.05.

## Results

### The correlation of PRMT4 expression and patient clinicopathological features and survival

**Figure 1:**
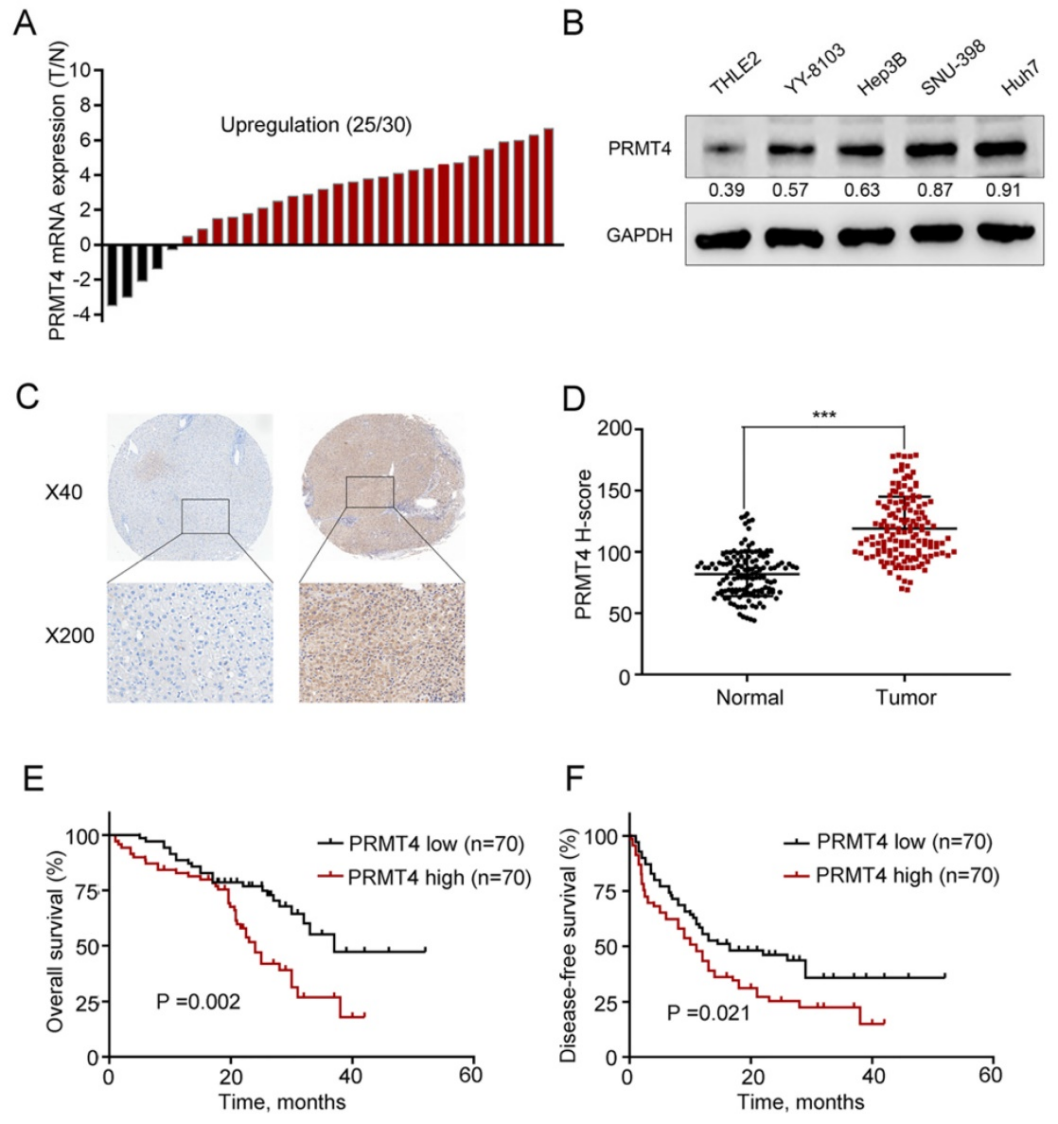
** PRMT4 expression is upregulated in HCC tissues and correlated with the clinical prognosis**. (A) Waterfall plot analyses of PRMT4 mRNA levels in 30 pairs of tumor tissues (T) and adjacent nontumor tissues (N) by qRT-PCR. (B) The protein level of PRMT4 in HCC cell lines was examined by Western blot. (C) PRMT4 immunostaining of tissue microarray comprising 140 pairs of HCC tissue and corresponding nontumor tissues. Representative immunostaining images of PRMT4 expression. (D) The H-score of PRMT4 staining in 140 pairs of HCC tissues and corresponding nontumor tissues. (E), (F) Kaplan-Meier analysis of overall survival (E) and disease-free survival (F) of tissue microarray data containing 140 patients. ^***^P < 0.001.

First, we collected 30 pairs of HCC tissues and their matched nontumor counterparts and analyzed the expression level of PRMT4 at the mRNA level using qRT-PCR, and the results showed upregulation of PRMT4 in 83.3% (25 of 30) of the HCC specimens compared with their nontumor counterparts (Figure 1A). Furthermore, PRMT4 expression levels were compared between the noncancerous THLE2 liver cell line and the 4 HCC cell lines, and Western blot analysis showed that PRMT4 expression was significantly higher in HCC cell lines than in the normal liver cell line (Figure 1B). Moreover, to further explore the clinical significance of PRMT4 in patients with HCC, the expression of PRMT4 in a TMA containing 140 pairs of HCC specimens and adjacent nontumor tissues was detected by immunohistochemical staining. Immunohistochemical staining demonstrated a higher expression density of PRMT4 in HCC tissues than in adjacent nontumor tissues. Moreover, in HCC tissues, positive PRMT4 staining was observed in both the nucleus and cytoplasmin, whereas in adjacent liver tissue, weak positive staining of PRMT4 was observed in the cytoplasm (Figure 1C). In addition, the H-scores were significantly higher in the HCC tissues than in the adjacent nontumor tissues (P <0.001, Figure 1D). Based on the median H-score of HCC tissue samples, the 140 patients were divided into the following two groups: PRMT4 low (score < 114, 70 patients) and PRMT4 high (score ≥ 114, 70 patients). Subsequent Kaplan-Meier analysis revealed that the PRMT4 high group had a poorer overall survival (P =0.002, Figure 1E) and disease-free survival (P =0.021, Figure 1F) than the PRMT4 low group. Furthermore, the relationship between PRMT4 expression and clinicopathologic features was analyzed in this cohort of HCC patients. As shown in Table 1, alpha-fetoprotein (AFP) levels (*P* = 0.040), tumor size (*P* = 0.006), satellite nodules (*P* = 0.024), and microvascular invasion (*P*<0.001) were significant parameters that correlated with the expression of PRMT4. Cox's multivariate proportional hazards model identified some risk factors for overall survival (Table 2): AFP ≥ 20 ng/mL (hazard ratio (HR): 2.121, 95% confidence interval (CI): 1.364-3.032, *P* = 0.008); tumor size ≥ 5 cm (HR: 2.998, 95% CI: 1.841-3.798, *P* = 0.001); microvascular invasion (HR: 2.125, 95% CI: 1.451-2.945, *P* < 0.001); and high PRMT4 expression (HR: 1.985, 95% CI: 1.451-3.754, *P* = 0.003). The risk factors for disease-free survival were as follows (Table 3): tumor size ≥ 5 cm (HR: 2.754, 95% CI: 1.564-3.564, *P* = 0.012); microvascular invasion (HR: 2.012, 95% CI: 1.398-2.874, *P* = 0.002); and high PRMT4 expression (HR: 2.124, 95% CI: 1.654-3.812, *P* = 0.006). Taken together, these results revealed that PRMT4 was upregulated in clinical HCC tissues and cell lines, and its overexpression correlated with the poor prognosis of HCC patients.

### PRMT4 overexpression promotes the proliferation, migration, and invasion of HCC cells

**Figure 2:**
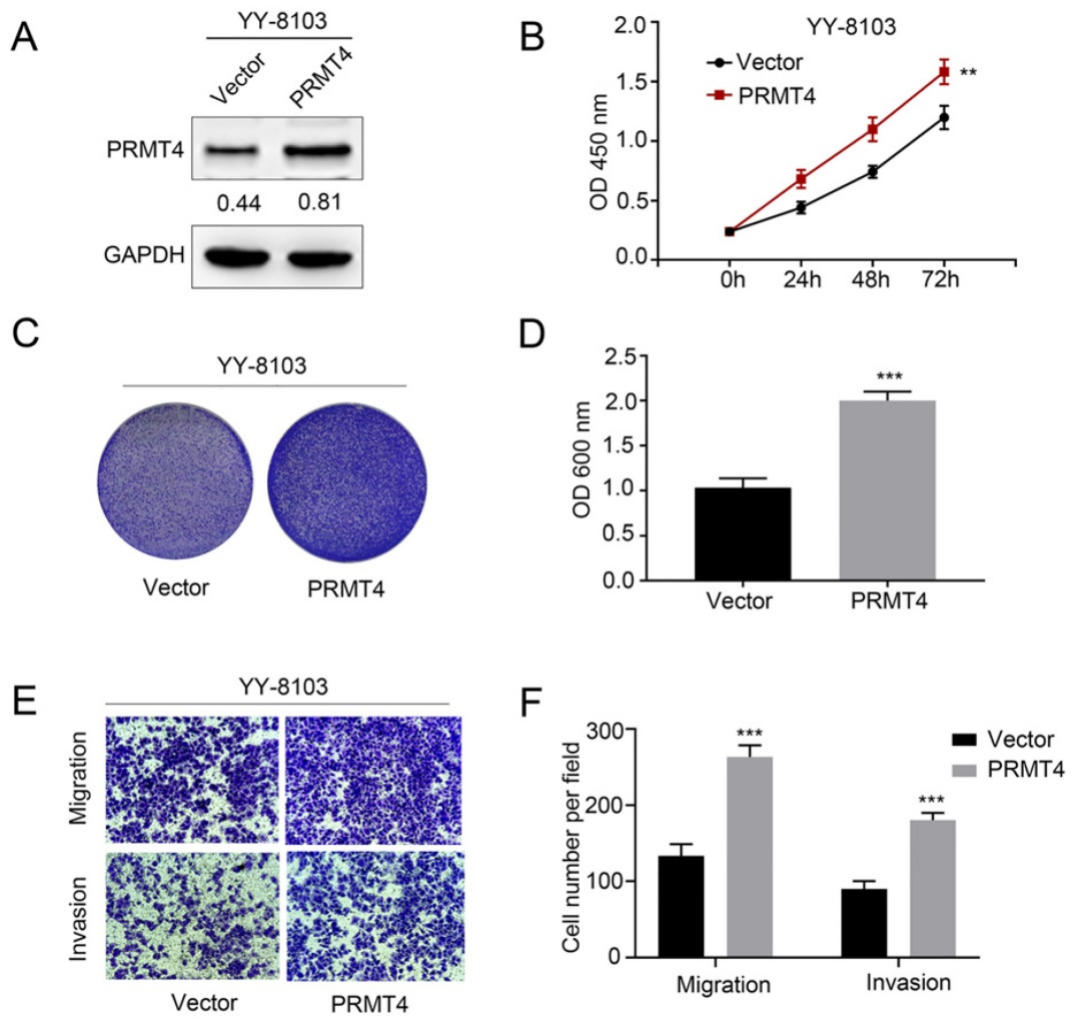
** PRMT4 overexpression promotes proliferation, migration, and invasion of HCC cells *in vitro***. (A) Western blots showing the overexpression of PRMT4 in YY-8103 cells. (B) The effect of PRMT4 overexpression on the proliferation of YY-8103 was measured by CCK-8 assays. (C) The effect of PRMT4 overexpression on the proliferation of YY-8103 cells was detected by crystal violet assays. (D) The OD value of crystal violet assays in YY-8103 cells. (E) The effect of PRMT4 overexpression on the migration and invasion of YY-8103 cells was detected by Transwell assays (×400 magnification). (F) Calculation of cells that migrated and invaded through the filter in YY-8103 cells. Measurement data were expressed as mean ± standard deviation (SD), and the experiments were repeated at least 3 times. ^**^P < 0.01, ^***^P < 0.001 *vs* Vector group.

Due to the close correlation between PRMT4 upregulation and tumor size and microvascular invasion, the aforementioned clinical observations encouraged us to investigate the biological function of PRMT4 in HCC cells, including its effects on proliferation, migration, and invasion. First, we exogenously overexpressed PRMT4 in YY-8103 cells, which showed relatively low expression levels of exogenous PRMT4 (Figure 2A). Then, we explored the effect of exogenous PRMT4 overexpression on cellular growth using CCK-8 and crystal violet assays. Indeed, the CCK-8 assay showed that the absorbance values of the YY-8103 cells after transfection with PRMT4 overexpression vectors were significantly higher than those of the vector controls (P < 0.01, Figure 2B). Furthermore, our results showed that high PRMT4 expression markedly induced the proliferation and colony formation of YY-8103 cells (P < 0.01, Figure 2B-D). Moreover, Transwell assays demonstrated that high PRMT4 expression remarkably increased the migration and invasion capabilities of YY-8103 cells (all P < 0.001, Figure 2E and F). Taken together, the results above indicated that PRMT4 overexpression induced the proliferation, migration and invasion of HCC cell lines.

### Silencing PRMT4 inhibits the proliferation, migration, and invasion of HCC cells

**Figure 3:**
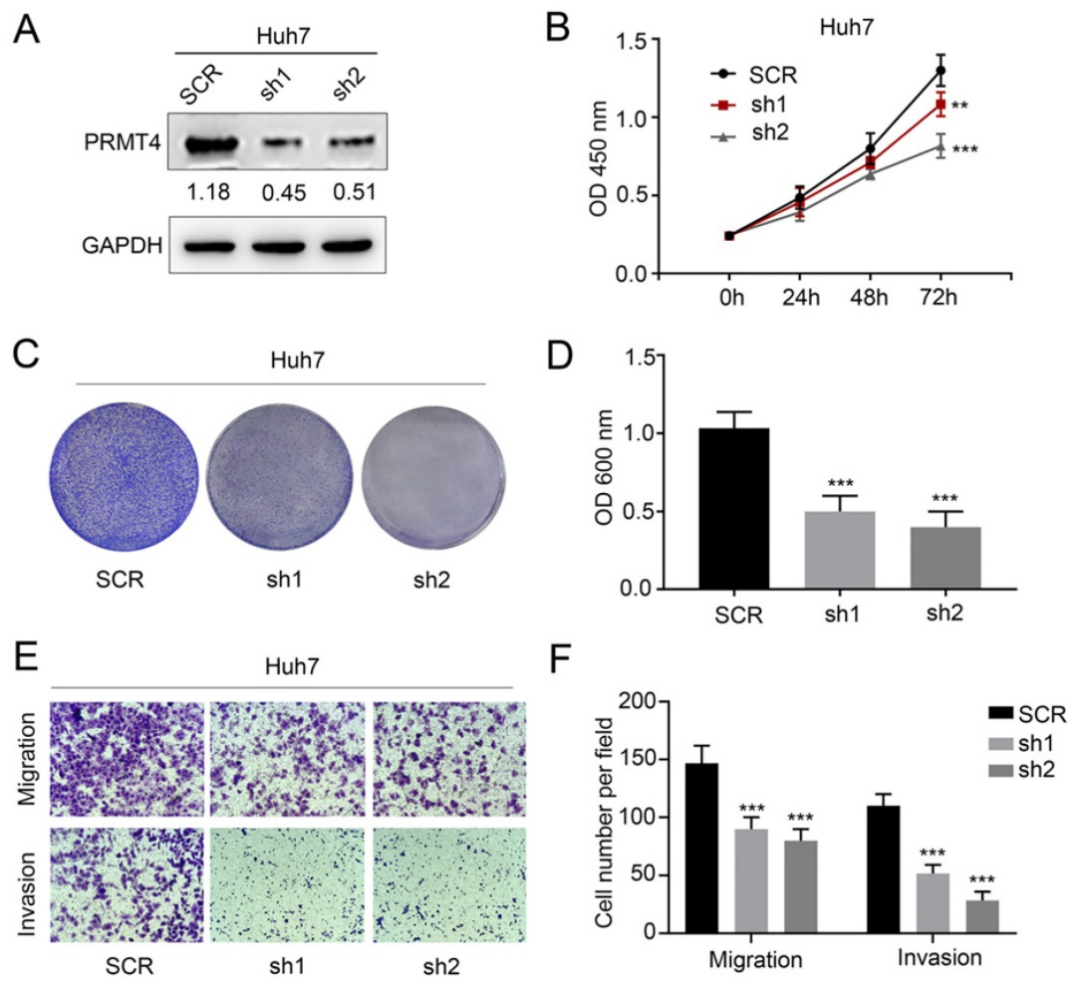
** PRMT4 knockdown inhibits proliferation, migration, and invasion of HCC cells *in vitro***. (A) Western blots showing the downregulation of PRMT4 in Huh7 cells. (B) The effect of PRMT4 knockdown on the proliferation of Huh7 was measured by CCK-8 assays. (C) The effect of PRMT4 knockdown on the proliferation of Huh7 cells was detected by crystal violet assays. (D) The OD value of crystal violet assays in Huh7 cells. (E) The effect of PRMT4 knockdown on the migration and invasion of Huh7 cells was detected by Transwell assays (×400 magnification). (F) Calculation of cells that migrated and invaded through the filter in Huh7 cells. Measurement data were expressed as mean ± SD, and the experiments were repeated at least 3 times. ^**^P < 0.01, ^***^P < 0.001 *vs* SCR group. SCR, scrambled; shRNA, short hairpin RNA.

We successfully downregulated the expression of PRMT4 by two targeted shRNAs in Huh7 cell lines, which showed relatively high expression levels of exogenous PRMT4 (Figure 3A). Similarly, we tested the proliferation, migration, and invasion of the control and PRMT4-shRNA cell lines. Based on CCK-8 (Figure 3B) and crystal violet assay results (Figure 3C and D), we found that PRMT4 knockdown inhibited the proliferation and colony formation of Huh7 cells. Moreover, the Transwell assay results demonstrated that PRMT4 downregulation markedly suppressed the invasion and migration capabilities of Huh7 cells (Figure 3E and F). Altogether, these findings indicated that downregulation of PRMT4 expression may exert a protective effect by suppressing the proliferation, migration and invasion of HCC cell lines.

### PRMT4 induces the progression of HCC cells by activating the AKT/mTOR pathway

**Figure 4:**
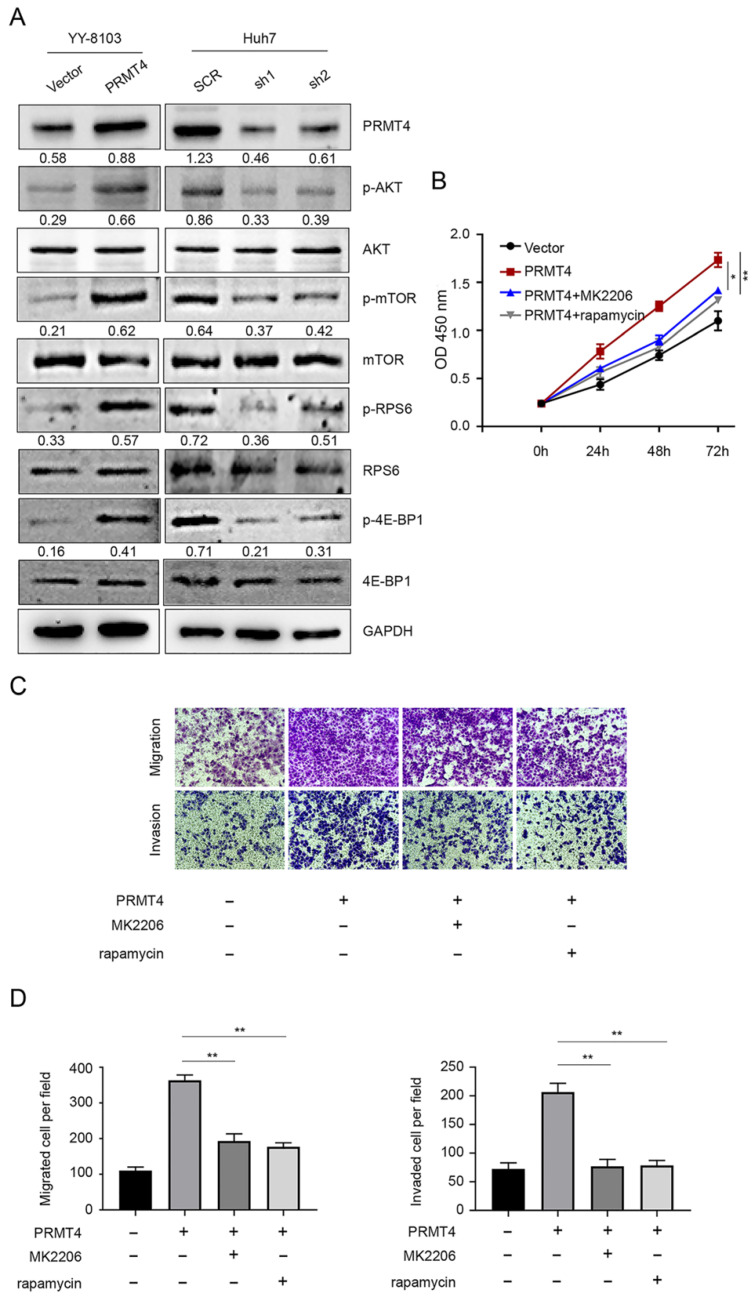
** PRMT4 positively regulates the AKT/mTOR signaling pathway**. (A) The expression levels of AKT, p-AKT, mTOR, p-mTOR, RPS6, p-RPS6, 4E-BP1 and p-4E-BP1 were detected by western blot in stable cell lines. (B) The inhibition of the AKT/mTOR pathway abolished the cell proliferation mediated by PRMT4 overexpression, as indicated by CCK-8 assays. (C) The inhibition of the AKT/mTOR pathway abolished the cell migration and invasion mediated by PRMT4 overexpression, as indicated by Transwell assays (×400 magnification). (D) Calculation of cells that migrated and invaded through the filter in YY-8103 cells. Measurement data were expressed as mean ± SD, and the experiments were repeated at least 3 times. *P < 0.05, **P < 0.01vs PRMT4 group.

Constitutive AKT/mTOR signaling pathway activation is known to play a critical role in the development of HCC 21. We therefore hypothesized that PRMT4 might promote HCC progression through the AKT/mTOR pathway. Western blotting analysis was carried out to investigate the activation of the AKT/mTOR signaling pathway in PRMT4 overexpression and knockdown cell lines. In YY-8103 and Huh7 cells, overexpression of PRMT4 increased, whereas silencing of PRMT4 decreased the levels of p-AKT, p-mTOR, p-RPS6 and p-4EBP1; while, the levels of AKT, mTOR, RPS6 and 4EBP1 were not significantly changed (Figure 4A). These results indicated that PRMT4 probably exerted its oncogenic role in HCC cells by activating the AKT/mTOR pathway. To further elucidate whether AKT/mTOR plays a specific role in PRMT4-mediated biological processes in HCC cells, rescue experiments were conducted using a specific inhibitor of AKT (1 μM MK2206) or mTOR (100 nM rapamycin). Significantly, as shown by the CCK-8 assay results, MK2206 and rapamycin attenuated the cell proliferation induced by PRMT4 overexpression (Figure 4B). As shown in Figure 4C and D, the migration and invasion ability of PRMT4-infected cells was inhibited by MK2206 or LY294002, as indicated by Transwell assays. These findings indicated that PRMT4 exerted its oncogenic role in HCC progression via activation of the AKT/mTOR pathway.

## Discussion

In this study, we first identified a novel oncogene, PRMT4, whose overexpression predicts poor prognosis for HCC patients. Second, we observed that PRTM4 overexpression could promote the proliferation, migration, and invasion of HCC cells *in vitro*. Third, we determined that the PRMT4-related tumor-promoting effect in HCC may depend on the activation of the AKT/mTOR pathway.

PRMT4 was first identified as a protein with arginine-specific histone methyltransferase activity 22. As a type I PRMT, PRMT4 catalyzes the formation of ADMA and MMA. Previous studies have shown that PRMT4 function is dysregulated in cancer. However, PRMT4 function is complex and context-dependent in cancer development and PRMT4 acts as both a tumor suppressor and as a tumor-promoting protein. For example, Liu et al. revealed that PRMT4 can maintain LSD1 stability by promoting the binding between deubiquitinase USP7 and LSD1, thereby promoting migration and invasion of breast cancer cells and facilitating cancer metastasis 23. However, Dhaheri et al. reported that PRMT4, as a coactivator of the estrogen receptor ERα, inhibits estrogen-dependent breast cancer cell proliferation and induces differentiation 24. In addition, Kim et al. showed that PRMT4 is particularly overexpressed in colorectal cancer and exerts a biological effect by regulating p53 and NF-κB target gene expression 20. Wu et al. found that the expression of PRMT4 is elevated in non-small cell lung cancer (NSCLC), and silencing PRMT4 could reduce the proliferative activity of NSCLC cells (PC9 and HCC827), suggesting an oncogenic role for PRMT4 in NSCLC 17. In contrast, Hu et al. illustrated that overexpression of PRMT4 inhibits the proliferation and invasion of lung cancer cells (H1299 and PC14) and promotes lung cancer cell apoptosis and is negatively correlated with the degree of tumor metastasis, which indicates a tumor suppressor role for PRMT4 in lung cancer 25. Importantly, in liver cancer, Osada et al. indicated that the expression of PRMT4 during hepatocellular carcinogenesis is increased in adenomas and is aberrant in carcinomas 26. Similarly, Shin et al. found that the nuclear expression and the rate of nuclear localization of PRMT4 are significantly higher in cancerous regions than in noncancerous regions and that the AMPK-ERK/PRMT4 autophagy signaling pathway is involved in liver cancer formation 27. Inversely, Zhong et al. recently found that PRMT4 expression is decreased in malignant liver cancer and methylates GAPDH at R234 to repress glycolysis and proliferation of liver cancer cells, indicative of a tumor suppressor role 28. Taken together, it is reasonable to conclude that PRMT4 can function as either a tumor-promoting or antiproliferative protein, suggesting that the actions of PRMT4 might depend on the cellular context and tumor type. Moreover, tumors show wide heterogeneity in terms of molecular expression characteristics, and gene profiling studies of tumors have identified specific subtypes with clinical and biological importance. A previous study indicated that PRMT4 is associated with the histological type of breast cancer and is less frequently expressed in lobular and tubular types, and a positive correlation was found between PRMT4 expression and biomarkers associated with a nonluminal phenotype and poor prognosis, such as HER-2, basal cytokeratins, and the proliferation marker Ki67 18. Thus, large-scale cohort studies and detailed immunohistochemical analysis of PRMT4 in cancer samples will be invaluable to better understand the role PRMT4 plays in liver cancer progression. The prognostic value of PRMT4 expression also merits future investigation. In the present study, we found that PRMT4 was highly expressed in HCC tumor tissues compared to adjacent nontumor tissues. Additionally, the tissue microarray assay revealed that PRMT4 was associated with tumor-initiating clinicopathologic variables and acted as an independent and significant risk factor for survival and recurrence. Next, biological functional experiments showed that PRMT4 overexpression could promote HCC cell proliferation, migration, and invasion *in vitro*, while knocking down PRMT4 inhibited these tumor characteristics. These findings revealed that PRMT4 may be a reliable biomarker to predict prognosis for HCC patients.

To explore the underlying mechanism of PRMT4 in HCC, we evaluated the effect of PRMT4 on the AKT/mTOR signaling pathway. The AKT/mTOR signaling pathway is a major signaling pathway that is activated in human malignancies and regulates multiple cellular processes, such as cell proliferation, apoptosis and migration 21, 29. AKT activation promotes metastasis and invasion of cancer cells and phosphorylates mammalian target of rapamycin (mTOR) 30. mTOR is an important downstream target of AKT and phosphorylates 70S6K and 4E-BP1 to stimulate protein synthesis 31. The phosphorylation of RPS6 induced by p70S6K activation ultimately drives the translation of 5'TOP (terminal oligopyrimidine tract) mRNAs 32. 4E-BP1 is a translation inhibitor that binds to the translation initiation factor eIF4E. Upon growth signaling, 4E-BP1 is phosphorylated and inactivated, leading to the release of eIF4E from 4E-BP1. Free eIF4E binds to the cap structure and promotes cap-dependent mRNA translation 33. Previous studies have demonstrated frequent changes in the AKT/mTOR pathway in HCC 34-36. Components of this pathway are frequently dysregulated in an extensive number of tumors, making the AKT/mTOR signaling pathway an attractive target for cancer therapy 21, 37. Increasing evidence indicates that inhibition of the AKT/mTOR pathway suppresses cell growth in many tumor types 38, 39. Previous studies have reported that PRMTs exert their biological functions through the regulation of the AKT/mTOR pathway. PRMT6 behaves as an oncogene to promote cell proliferation and migration in endometrial cancer via activation of the AKT/mTOR pathway 40. PRMT5 promotes human lung cancer cell proliferation via direct interaction and regulation of AKT activation 41. Importantly, PRMT4 specifically catalyzes H3R26me2 in porcine embryos and participates in blastocyst development by regulating key genes associated with the PI3K-AKT signaling pathway 42. In experiments investigating the underlying mechanism, we found that the expression levels of p-AKT, p-mTOR, p-RPS6 and p-4E-BP1 were markedly increased following PRMT4 overexpression, which indicated that PRMT4 may play an oncogenic role in HCC cells by activating the AKT/mTOR pathway. Furthermore, inhibition of AKT/mTOR signaling by MK2206 or rapamycin significantly attenuated the PRMT4-mediated malignant phenotypes. Since inhibitors of AKT/mTOR represent potential agents in human cancers, the newly identified PRMT4/AKT/mTOR axis may serve as both a prognostic factor and therapeutic target in HCC.

## Conclusion

In summary, this study demonstrated that PRMT4 overexpression was associated with aggressive tumor behaviors and a poor prognosis in patients with HCC. PRMT4 promotes HCC progression by activating the AKT/mTOR signaling pathway. Thus, PRMT4 may serve as a valuable biomarker and potential target for HCC.

## Figures and Tables

**Table 1:** Relationship between the PRMT4 expression and the clinicopathologic features of HCC.

Variables	Total (n=140)	PRMT4 expression, n (%)		*P*-value
		Low (n=70)	High (n=70)	
Sex				0.421
Male	108	56 (80.0)	52 (74.3)	
Female	32	14 (20.0)	18 (25.7)	
Age, year				0.608
<60	59	31 (44.3)	28 (40.0)	
≥60	81	39 (55.7)	42 (60.0)	
HBsAg				0.438
Negative	17	10 (14.3)	7 (10.0)	
Positive	123	60 (85.7)	63 (90.0)	
AFP, ng/ml				**0.040**
<20	60	36 (51.4)	24 (34.3)	
≥20	80	34 (48.6)	46 (65.7)	
Tumor number				0.303
Single	82	38 (54.3)	44 (62.9)	
Multiple	58	32 (45.7)	26 (37.1)	
Tumor size, cm				**0.006**
<5	43	29 (41.4)	14 (20.0)	
≥5	97	41 (58.6)	56 (80.0)	
Satellite nodules				**0.024**
No	85	49 (70.0)	36 (51.4)	
Yes	55	21 (30.0)	34 (48.6)	
Lymph node invasion				0.629
No	120	61 (87.1)	59 (84.3)	
Yes	20	9 (12.9)	11 (15.7)	
Microvascular invasion				**<0.001**
Negative	82	55 (81.3)	27 (38.6)	
Positive	58	15 (18.8)	43 (61.4)	

**Abbreviation**: HBsAg: Hepatitis B surface antigen; AFP: α -fetoprotein. Bold denotes *P*-value <0.05.

**Table 2:** Univariate and multivariate cox regression analyses of overall survival.

Characteristic	Univariate				Multivariate		
	HR (95% CI)	*P*-value				HR (95% CI)	*P*-value
Sex, male vs. female	1.128(0.885-1.421)	0.521					
Age≥60, year	1.132(0.898-1.512)	0.312					
HBsAg, positive	1.144(0.804-1.630)	0.365					
AFP, ≥20 ng/ml	2.369(1.451-3.125)	**0.001**				2.121(1.364-3.032)	**0.008**
Tumor number, multiple vs. single	1.125(0.645-2.585)	0.195					
Tumor size, ≥5 cm	3.122(1.951-3.954)	**<0.001**				2.998(1.841-3.798)	**0.001**
Satellite nodules, yes	1.594(0.931-1.894)	0.098					
Lymph node invasion, yes	1.265(0.894-1.541)	0.225					
Microvascular invasion, positive	2.351(1.564-3.120)	**<0.001**				2.125(1.451-2.945)	**<0.001**
PRMT4 expression, high	2.011(1.651-3.854)	**<0.001**				1.985(1.451-3.754)	**0.003**
							

**Abbreviation**: HBsAg: Hepatitis B surface antigen; AFP: α -fetoprotein. Bold denotes *P*-value <0.05.

**Table 3:** Univariate and multivariate cox regression analyses of disease-free survival.

Characteristic	Univariate				Multivariate		
	HR (95% CI)	*P*-value				HR (95% CI)	*P*-value
Sex, male vs. female	1.111(0.854-1.395)	0.454					
Age≥60, year	1.232(0.912-1.601)	0.412					
HBsAg, positive	1.214(0.854-1.652)	0.254					
AFP, ≥20 ng/ml	2.456(1.598-3.384)	0.112					
Tumor number, multiple vs. single	1.119(0.612-2.531)	0.154					
Tumor size, ≥5 cm	2.945(1.754-3.845)	**0.001**				2.754(1.564-3.564)	**0.012**
Satellite nodules, yes	1.654(0.954-2.018)	0.111					
Lymph node invasion, yes	1.335(0.789-2.017)	0.385					
Microvascular invasion, positive	2.151(1.433-3.011)	**<0.001**				2.012(1.398-2.874)	**0.002**
PRMT4 expression, high	2.549(1.798-3.987)	**0.001**				2.124(1.654-3.812)	**0.006**
							

**Abbreviation**: HBsAg: Hepatitis B surface antigen; AFP: α -fetoprotein. Bold denotes *P*-value <0.05

## References

[1] Sung H, Ferlay J, Siegel RL, Laversanne M, Soerjomataram I, Jemal A (2021). Global Cancer Statistics 2020: GLOBOCAN Estimates of Incidence and Mortality Worldwide for 36 Cancers in 185 Countries. CA Cancer J Clin.

[2] Yang JD, Heimbach JK (2020). New advances in the diagnosis and management of hepatocellular carcinoma. Bmj.

[3] Yu WB, Rao A, Vu V, Xu L, Rao JY, Wu JX (2017). Management of centrally located hepatocellular carcinoma: Update 2016. World J Hepatol.

[4] Hu J, Gao DZ (2012). Distinction immune genes of hepatitis-induced heptatocellular carcinoma. Bioinformatics.

[5] Marquardt JU, Galle PR, Teufel A (2012). Molecular diagnosis and therapy of hepatocellular carcinoma (HCC): an emerging field for advanced technologies. J Hepatol.

[6] Yang Y, Bedford MT (2013). Protein arginine methyltransferases and cancer. Nat Rev Cancer.

[7] Hwang JW, Cho Y, Bae GU, Kim SN, Kim YK (2021). Protein arginine methyltransferases: promising targets for cancer therapy. Exp Mol Med.

[8] Yoshimatsu M, Toyokawa G, Hayami S, Unoki M, Tsunoda T, Field HI (2011). Dysregulation of PRMT1 and PRMT6, Type I arginine methyltransferases, is involved in various types of human cancers. Int J Cancer.

[9] Shailesh H, Zakaria ZZ, Baiocchi R, Sif S (2018). Protein arginine methyltransferase 5 (PRMT5) dysregulation in cancer. Oncotarget.

[10] Song C, Chen T, He L, Ma N, Li JA, Rong YF (2020). PRMT1 promotes pancreatic cancer growth and predicts poor prognosis. Cell Oncol (Dordr).

[11] Stopa N, Krebs JE, Shechter D (2015). The PRMT5 arginine methyltransferase: many roles in development, cancer and beyond. Cell Mol Life Sci.

[12] Chan LH, Zhou L, Ng KY, Wong TL, Lee TK, Sharma R (2018). PRMT6 Regulates RAS/RAF Binding and MEK/ERK-Mediated Cancer Stemness Activities in Hepatocellular Carcinoma through CRAF Methylation. Cell Rep.

[13] Jiang H, Zhou Z, Jin S, Xu K, Zhang H, Xu J (2018). PRMT9 promotes hepatocellular carcinoma invasion and metastasis via activating PI3K/Akt/GSK-3β/Snail signaling. Cancer Sci.

[14] Miranda TB, Miranda M, Frankel A, Clarke S (2004). PRMT7 is a member of the protein arginine methyltransferase family with a distinct substrate specificity. J Biol Chem.

[15] Covic M, Hassa PO, Saccani S, Buerki C, Meier NI, Lombardi C (2005). Arginine methyltransferase CARM1 is a promoter-specific regulator of NF-kappaB-dependent gene expression. Embo j.

[16] An W, Kim J, Roeder RG (2004). Ordered cooperative functions of PRMT1, p300, and CARM1 in transcriptional activation by p53. Cell.

[17] Wu D, He J, Zhang W, Wang K, Jin S, Li J (2020). CARM1 promotes non-small cell lung cancer progression through upregulating CCNE2 expression. Aging (Albany NY).

[18] Habashy HO, Rakha EA, Ellis IO, Powe DG (2013). The oestrogen receptor coactivator CARM1 has an oncogenic effect and is associated with poor prognosis in breast cancer. Breast Cancer Res Treat.

[19] Zheng L, Chen J, Zhou Z, He Z (2017). miR-195 enhances the radiosensitivity of colorectal cancer cells by suppressing CARM1. Onco Targets Ther.

[20] Kim YR, Lee BK, Park RY, Nguyen NT, Bae JA, Kwon DD (2010). Differential CARM1 expression in prostate and colorectal cancers. BMC Cancer.

[21] Aoki M, Fujishita T (2017). Oncogenic Roles of the PI3K/AKT/mTOR Axis. Curr Top Microbiol Immunol.

[22] Lee YH, Koh SS, Zhang X, Cheng X, Stallcup MR (2002). Synergy among nuclear receptor coactivators: selective requirement for protein methyltransferase and acetyltransferase activities. Mol Cell Biol.

[23] Liu J, Feng J, Li L, Lin L, Ji J, Lin C (2020). Arginine methylation-dependent LSD1 stability promotes invasion and metastasis of breast cancer. EMBO Rep.

[24] Al-Dhaheri M, Wu J, Skliris GP, Li J, Higashimato K, Wang Y (2011). CARM1 is an important determinant of ERα-dependent breast cancer cell differentiation and proliferation in breast cancer cells. Cancer Res.

[25] Hu B, Li X, Chen L, Liu Z (2020). High Expression of CARM1 Inhibits Lung Cancer Progression by Targeting TP53 by Regulating CTNNB1. Lung.

[26] Osada S, Suzuki S, Yoshimi C, Matsumoto M, Shirai T, Takahashi S (2013). Elevated expression of coactivator-associated arginine methyltransferase 1 is associated with early hepatocarcinogenesis. Oncol Rep.

[27] Shin HJ, Kim H, Oh S, Lee JG, Kee M, Ko HJ (2016). AMPK-SKP2-CARM1 signalling cascade in transcriptional regulation of autophagy. Nature.

[28] Zhong XY, Yuan XM, Xu YY, Yin M, Yan WW, Zou SW (2018). CARM1 Methylates GAPDH to Regulate Glucose Metabolism and Is Suppressed in Liver Cancer. Cell Rep.

[29] Alzahrani AS (2019). PI3K/Akt/mTOR inhibitors in cancer: At the bench and bedside. Semin Cancer Biol.

[30] Hua H, Kong Q, Zhang H, Wang J, Luo T, Jiang Y (2019). Targeting mTOR for cancer therapy. J Hematol Oncol.

[31] Cornu M, Albert V, Hall MN (2013). mTOR in aging, metabolism, and cancer. Curr Opin Genet Dev.

[32] García-Maceira P, Mateo J (2009). Silibinin inhibits hypoxia-inducible factor-1alpha and mTOR/p70S6K/4E-BP1 signalling pathway in human cervical and hepatoma cancer cells: implications for anticancer therapy. Oncogene.

[33] Bjornsti MA, Houghton PJ (2004). The TOR pathway: a target for cancer therapy. Nat Rev Cancer.

[34] Ma L, Ji L, Yu Y, Wang J (2015). Novel molecular targets for diagnosis and treatment of hepatocellular carcinoma. Discov Med.

[35] Luo X, Cao M, Gao F, He X (2021). YTHDF1 promotes hepatocellular carcinoma progression via activating PI3K/AKT/mTOR signaling pathway and inducing epithelial-mesenchymal transition. Exp Hematol Oncol.

[36] Wu Y, Zhang Y, Qin X, Geng H, Zuo D, Zhao Q (2020). PI3K/AKT/mTOR pathway-related long non-coding RNAs: roles and mechanisms in hepatocellular carcinoma. Pharmacol Res.

[37] Camillo Porta, Chiara Paglino, Alessandra Mosca (2014). Targeting PI3K/Akt/mTOR Signaling in Cancer. Frontiers in Oncology.

[38] Eiden AM, Zhang S, Gary JM, Simmons JK, Mock BA (2016). Molecular Pathways: Increased Susceptibility to Infection Is a Complication of mTOR Inhibitor Use in Cancer Therapy. Clin Cancer Res.

[39] Papadimitrakopoulou V, Adjei AA (2006). The Akt/mTOR and mitogen-activated protein kinase pathways in lung cancer therapy. J Thorac Oncol.

[40] Jiang N, Li QL, Pan W, Li J, Zhang MF, Cao T (2020). PRMT6 promotes endometrial cancer via AKT/mTOR signaling and indicates poor prognosis. Int J Biochem Cell Biol.

[41] Zhang S, Ma Y, Hu X, Zheng Y, Chen X (2019). Targeting PRMT5/Akt signalling axis prevents human lung cancer cell growth. J Cell Mol Med.

[42] Cao Z, Tong X, Yin H, Zhou N, Zhang X, Zhang M (2021). Histone Arginine Methyltransferase CARM1-Mediated H3R26me2 Is Essential for Morula-to-Blastocyst Transition in Pigs. Front Cell Dev Biol.

