# PRMT4 promotes hepatocellular carcinoma progression by activating AKT/mTOR signaling and indicates poor prognosis: Erratum

**DOI:** 10.7150/ijms.71614

**Published:** 2022-03-03

**Authors:** Peng Du, Kaifeng Luo, Guoyong Li, Jisheng Zhu, Qi Xiao, Yong Li, Xingjian Zhang

**Affiliations:** Department of General Surgery, The First Affiliated Hospital of Nanchang University, Nanchang, Jiangxi 330006, China.

The original version of our article 1 unfortunately contained an error in Figure 3E. The picture of Huh7 cell invasion assay picture in the sh2 group in Figure 3E was incorrectly assembled. We sincerely apologize for this error. All authors were informed and approved the corrected figures. Figure 3 should be corrected as follows

## Figures and Tables

**Figure 3:**
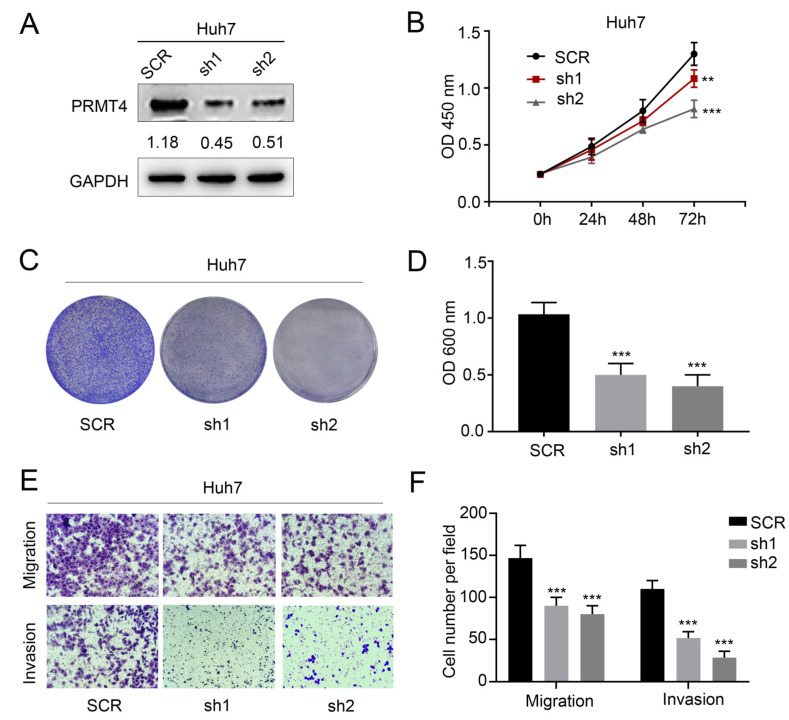
** PRMT4 knockdown inhibits proliferation, migration, and invasion of HCC cells in vitro**. (A) Western blots showing the downregulation of PRMT4 in Huh7 cells. (B) The effect of PRMT4 knockdown on the proliferation of Huh7 was measured by CCK-8 assays. (C) The effect of PRMT4 knockdown on the proliferation of Huh7 cells was detected by crystal violet assays. (D) The OD value of crystal violet assays in Huh7 cells. (E) The effect of PRMT4 knockdown on the migration and invasion of Huh7 cells was detected by Transwell assays (×400 magnification). (F) Calculation of cells that migrated and invaded through the filter in Huh7 cells. Measurement data were expressed as mean ± SD, and the experiments were repeated at least 3 times. ^**^P< 0.01, ^***^P < 0.001 vs SCR group. SCR, scrambled; shRNA, short hairpin RNA.
